# Differences in frequency between administrative and parent-reported ADHD diagnosis data of children and adolescents taking sociodemographic characteristics into account – Results from the consortium project INTEGRATE-ADHD

**DOI:** 10.25646/12674

**Published:** 2024-09-18

**Authors:** Robert Schlack, Ann-Kristin Beyer, Lilian Beck, Stefan Pfeifer, Heike Hölling, Thomas Jans, Annalena Berner, Vanessa Scholz, Sophia Weyrich, Anne Kaman, Ulrike Ravens-Sieberer, Julian Witte, Peter Heuschmann, Cordula Riederer, the INTEGRATE-ADHD Study Group, Marcel Romanos

**Affiliations:** 1 Robert Koch Institute, Department of Epidemiology and Health Monitoring, Berlin, Germany; 2 University Hospital Würzburg, Centre of Mental Health, Department of Child and Adolescent Psychiatry, Psychosomatics and Psychotherapy, Würzburg, Germany; 3 University Medical Centre Hamburg-Eppendorf, Department of Child and Adolescent Psychiatry, Psychotherapy and Psychosomatics, Research Section ‘Child Public Health’, Hamburg, Germany; 4 Vandage GmbH, Bielefeld, Germany; 5 University of Würzburg, Institute of Clinical Epidemiology and Biometry, Würzburg, Germany; 6 University Hospital Würzburg, Clinical Trial Centre, Würzburg, Germany; 7 University Hospital Würzburg, Institute for Medical Data Sciences, Würzburg, Germany; 8 DAK-Gesundheit, Hamburg, Germany

**Keywords:** ADHD, administrative, epidemiological, parent report, survey, prevalence, Data linkage, children, adolescents

## Abstract

**Background:**

In the project INTEGRATE-ADHD, administrative and parent-reported ADHD diagnosis data of children and adolescents were linked at person level for the first time in Germany. This contribution analyses discrepancies between the data sources, considering sociodemographic characteristics.

**Methods:**

Parents of 5,461 0- to 17-year-olds insured with the German statutory health insurance company DAK-Gesundheit in 2020, who had a confirmed administrative diagnosis of ADHD (ICD-10 F90.0-9) in at least one quarter (M1Q criterion), were surveyed online about their child’s ADHD diagnosis and other health and care-related topics. Using logistic regression, associations between the presence of a parental report of the child’s administrative ADHD diagnosis and sociodemographic predictors were analysed.

**Results:**

71.6 % of parents reported their child’s administrative diagnosis of ADHD in the survey. The diagnosis was significantly less likely to be reported by parents of girls, younger children, children with a migration background and children from nuclear families with both biological parents. There were no differences with regard to parental education, urbanisation (urban/rural) or density of care. Bivariate findings were confirmed in the multivariable model.

**Conclusions:**

Approximately one third of parents do not report their child’s administrative diagnosis of ADHD. The likelihood of parental reporting varies according to sociodemographic factors. This should be considered when contextualising the data sources in the future.

## 1. Introduction

Attention-deficit/hyperactivity disorder (ADHD) is one of the most commonly diagnosed mental disorders of childhood and adolescence, both in Germany and worldwide [1, 2]. It is characterised by the core symptoms inattention, impulsivity and hyperactivity and is associated with an increased risk of comorbid disorders (the co-occurrence of several mental disorders in one person), parental separation and divorce rates, substance use, risky traffic and accident behaviour, delinquency, lower school and educational success, and lower quality of life [3–8]. Schlander and colleagues [9] found that the direct costs of treating children and adolescents with ADHD were more than 2.5 times higher than for a control group matched for age, sex and membership of health insurance. The cost to statutory health insurance was estimated at 260 million euros in 2003. Hasemann et al. [10] report annual direct health-related additional costs of € 1,500 per child with an incident diagnosis of ADHD for statutory health insurance in 2020. Given the significant individual, social and economic consequences, ADHD is of considerable public health importance.

ADHD has often been the subject of controversial discussions in the past, focussed on issues such as the validity of diagnoses and its ‘true’ prevalence in the population. In the first decade of the millennium, for example, claims data (Infobox) from various statutory health insurance funds revealed a sharp increase in the prevalence of ADHD diagnoses and a rising prevalence of prescriptions for ADHD medications in children and adolescents, particularly methylphenidate. Based on data of the statutory health insurance company AOK from the German state of Hesse, Schubert and colleagues found a 53 % increase in diagnosis prevalence (from 1.52 % to 2.21 %) in children and adolescents aged 5 to 18 years between 2000 and 2007 [11]. Based on nationwide data from Barmer-GEK, Grobe et al. [12, 13] reported an increase in the administrative prevalence of ADHD of 49 % (from 2.9 % to 4.1 %) between 2006 and 2011 for the age group 0 to 19 years, while Bachmann et al. [14] reported an increase from 5.0 % to 6.1 % between 2009 and 2014 for 0- to 17-year-olds based on nationwide AOK data. Increases in diagnosis prevalence have also been reported from outpatient claims data of the National Association of Statutory Health Insurance Physicians (KBV, from 3.7 % to 4.4 % in 5- to 14-year-olds between 2008 and 2011) [15]. Although the administrative prevalence rates have recently stagnated at high levels [16, 17], the trend was considered a cause for concern. For example, a special report by the German Advisory Council on the Assessment of Developments in the Health Care System in 2009 described ADHD as a ‘fashion diagnosis’ [18]. Also, the amendment of the drug guideline of the German Federal Joint Committee (G-BA) in 2009/2010 to restrict the prescription of methylphenidate for the treatment of children and adolescents with ADHD [19], calling simultaneously for more careful and guideline-conform diagnosis, is to be seen in this context.

**Infobox**

**Administrative data**

Administrative data is generated as part of administrative procedures. Important data sources for health reporting purposes are the claims data of statutory health insurance funds, from which, for example, prevalence rates (frequencies) of billed medical or psychological diagnoses can be determined. In addition, this data includes information on age and gender of the insured persons, on the utilisation of various outpatient and inpatient healthcare services, drug prescription data and information on the direct costs of utilisation. The administrative diagnostic data on ADHD of children and adolescents used in the project INTEGRATE-ADHD relates to the year 2020 and stems from the statutory health insurance provider DAK-Gesundheit.

**Epidemiological data**

Epidemiological data is collected through surveys and examinations with the aim of researching the prevalence and causes of diseases in the population. Frequencies of diagnosed physical diseases and mental disorders are often assessed asking the participants whether a doctor (or a psychologist) had diagnosed the respective disease/disorder. The diagnostic data on ADHD in children and adolescents collected in the online survey of the project INTEGRATE-ADHD is based on the parents’ report of an ADHD diagnosis of their child ever made by a medical doctor or psychologist. In addition, the epidemiological data collected in the project INTEGRATE-ADHD also includes questions on sociodemographics (e.g. age and gender of the child, parental education, history of migration), psychopathology and comorbidity (e.g. ADHD symptom severity, ADHD diagnosis of the parents, anxiety, depression), risk and protective factors, quality of life, as well as satisfaction with care and barriers to utilisation.

Population-based figures on the prevalence of ADHD for Germany, however, are not only available from the claims data of the statutory health insurance funds or the KBV, but also from the epidemiological German Health Interview and Examination Survey for Children and Adolescents (KiGGS) conducted by the Robert Koch Institute [20–24] and its in-depth module on child mental health, the BELLA study [25]. In the KiGGS study, parents of 3- to 17-year-old children and adolescents were asked whether their child had ever been received a dotctor’s or psychologist’s ADHD diagnosis. The parent-reported diagnosis rates recorded in this way remained constant at around 5 % over a period comparable to that in which the last significant increases in prevalence were reported (i.e., from the KiGGS baseline study 2003 – 2006 to KiGGS Wave 1 2009 – 2011) [23]. In KiGGS Wave 2 (survey period 2014 – 2017), the parent-reported frequency of ADHD diagnosis even decreased by 0.9 percentage points (from 5.3 % to 4.4 %, or by about 17 %) compared with the baseline study [20, 24].

The administrative and epidemiological data on the frequency of ADHD diagnoses in Germany are therefore not consistent. Due to different data bases, different case definitions and inclusion criteria, different time references (annual prevalence in routine data vs. lifetime prevalence in the KiGGS study) or different reference populations (health insurance-specific population vs. general population), they are comparable only to a limited extent. Even though increasing efforts were made to relate the data sources to one an other in an interpretative way [20, 26, 27], there is a lack of studies that combine routine administrative data and epidemiologically collected survey data at the person level [26]. In addition, whether the underlying diagnosis was made according to the guidelines is unknown for either diagnoses data source. However, the issue of reliable prevalence figures and a valid diagnostics is of considerable importance to supply services most efficiently in the health care system and to avoid misallocation, both for those affected and for service providers and the community of insurees.

As part of the project INTEGRATE-ADHD, for the first time in Germany parents of statutorily insured children and adolescents with an administrative diagnosis of ADHD (health insurance company DAK-Gesundheit) were asked about their child’s ADHD diagnosis using the epidemiological questionnaires from the KiGGS and BELLA studies, and a sub-sample was examined with a guideline-based clinical examination [28, 29]. The project thus provides linkable administrative, epidemiological and clinical data on ADHD diagnosis at the person level.

In order to approximate the comparability of administrative and epidemiological data on ADHD diagnoses, the 12-month prevalence of a child’s ADHD had been recorded in the parent survey for the first time in KiGGS Wave 2 (2014 – 2017), in addition to the lifetime prevalence. A total of 2.8 % of parents of children and adolescents aged 3 to 17 years reported that their child had also had ADHD in the past twelve months [30]. Compared with the proportion of children and adolescents with an administrative diagnosis of ADHD in the DAK-insured population of 4.1 % [31], this would mean a discrepancy of 1.3 percentage points. It is therefore likely that a significant proportion of ADHD diagnoses are not reported by parents. This article examines how many parents report their child’s administrative diagnosis of ADHD in the epidemiological survey and how the parental reporting behaviour distributes across various sociodemographic characteristics. As this approach is, to the best of our knowledge, so far unique, no specific hypotheses were made regarding parental reporting frequencies in relation to different sociodemographic subgroups. These analyses were exploratory in nature.

## 2. Methods

### 2.1 Study design and conduct

The consortium project INTEGRATE-ADHD is a cross-sectional interview and examination study of parents with children and adolescents who had an administratively documented diagnosis of ADHD (ICD-10 F90.0-9) in at least one quarter of 2020 (so-called M1Q criterion). Parents were included if their children were insured with the third largest nationwide operating health insurance company in Germany, DAK-Gesundheit, in 2020, if they were aged 0 to 17 years at the time, and if the administrative diagnosis of ADHD was labelled as confirmed with the suffix ‘G’. The survey was conducted online from October 2021 to August 2022 using modified questionnaires from the nationwide epidemiological KiGGS study [32, 33] and its in-depth module on the mental health of children and adolescents, BELLA study [34, 35]. A sub-sample of children and adolescents were also assessed with an online clinical diagnostics according to the S3 guideline ADHD of the Association of the Scientific Medical Societies in Germany (in German: Arbeitsgemeinschaft der Wissenschaftlichen Medizinischen Fachgesellschaften, AWMF) [36].

Out of a total of *N* = 24,880 invited parents (gross sample), *n* = 5,919 parents completed the online survey. After excluding *n* = 458 individuals for formal and content reasons (including more than 50 % missing values or inconsistencies in age and gender information between the administrative and epidemiological data sets), the net sample was *n* = 5,461 participants. The response rate according to the American Association for Public Opinion Research (AAPOR) Standard Definitions, Version 9 (RR3) was 21.5 % [37]. From the group of children and adolescents whose parents agreed to participate in the online survey, *n* = 202 children and adolescents were randomly selected and clinically examined. For a description of the clinical assessment, see Hetzke et al. [38]. The data from the clinical sample are not the subject of this article. The online survey data, administrative data and clinical data were then linked at person level in order to form an integrated dataset (data linkage). The participating parents and, from the age of 14 years, the children and adolescents themselves consented to the data linkage after being informed in advance. Information from the online survey dataset and from the administrative data was included in this analysis.

#### Sample representativity and weighting

The children and adolescents insured with DAK-Gesundheit can be considered approximately representative of the population of children and adolescents in Germany in terms of gender and age. With regard to the population of children and adolescents with an administrative ADHD diagnosis, comparisons of the INTEGRATE gross sample with nationwide wide outpatient ADHD diagnosis data from the Central Research Institute of Ambulatory Health Care in Germany (Zi) from 2015 and 2016 [39] showed only very slight deviations in terms of distribution by gender, whereas younger children were overrepresented and older children and adolescents were underrepresented in the INTEGRATE ADHD gross sample [28].

Deviations of the net sample from the gross sample were adjusted using population weights, which normalise the net sample to the gross sample [38]. The population weights were determined by the inverse probability that an individual would participate in the study. People with a low probability of participation represent more of the population than people with a high probability of participation.

**ADHD in Germany – Comparison and integration of administrative and epidemiological ADHD diagnostic data through clinical assessment (INTEGRATE-ADHD)**

**Consortium partners:** Robert Koch Institute Berlin, Department of Epidemiology and Health Monitoring, Germany; University Hospital Würzburg, Department of Child and Adolescent Psychiatry, Psychosomatics and Psychotherapy, Germany; University Medical Centre Hamburg-Eppendorf, Department of Child and Adolescent Psychiatry, Psychotherapy and Psychosomatics, Research Section ‘Child Public Health’, Germany; Vandage GmbH, Germany; University of Würzburg, Germany; Institute for Clinical Epidemiology and Biometry, Germany; DAK-Gesundheit, Germany

**Data holder:** Robert Koch Institute

**Objectives:** Identification of potential causes for the discrepancies between administrative ADHD diagnostic data (based on health insurance claims data) and epidemiological ADHD diagnostic data (based on surveys) for Germany, integration and validation of these data through a guideline-based clinical examination

**Study design:** Cross-sectional online survey, additional clinical examination of a sub-sample, data linkage with administrative health insurance data

**Population:** Children and adolescents who were insured with DAK-Gesundheit in 2020 and who were 0 to 17 years old at that time and for whom an administrative ADHD diagnosis labelled as confirmed was available in at least one quarter

**Gross sample**: 24,880 children and adolescents insured with DAK-Gesundheit with an administrative ADHD diagnosis

**Net sample:** 5,461 surveyed parents, 202 clinically examined children and adolescents

**Data collection period:** October 2021 to August 2022 (online survey), January 2022 to January 2023 (online clinical examination)

More information in German at www.rki.de/integrate-adhd

### 2.2 Indicators

#### Parent-reported lifetime diagnosis of ADHD

According to the KiGGS case definition [22, 23], ADHD cases were considered valid if parents reported that their child had ever been diagnosed with ADHD or attention-deficit disorder (ADD) by a doctor or psychologist. Information was also considered valid if the parents had indicated institutions where a diagnosis could reasonybly expected to be made by medical or psychological staff (e.g. ‘university clinic’, ‘child and adolescent psychiatry’, ‘social paediatric centre’).

#### Sociodemographic indicators

##### Gender and age

Only binary gender information is available in the administrative data; in the online survey, the gender of 27 respondents, however, was reported as ‘diverse’. As this group was too small for statistical analysis, these individuals were assigned the gender information from the administrative data set. Where child gender was not provided in the survey, it was taken from the administrative data (*n* = 2). Gender was therefore included in the analyses with as girl/female and boy/male. Age was taken into account applying the developmental age group categorisation 0 to 2 years, 3 to 6 years, 7 to 10 years, 11 to 13 years, 14 to 17 years and 18 to 19 years, which were also used in the KiGGS study.

##### Parental educational status

As a proxy for socioeconomic status (SES), parental educational status was assessed according to the CASMIN (Comparative Analysis of Social Mobility in Industrial Nations) classification [40]. The CASMIN classification was developed to provide an internationally comparable classification of education. It includes information on both general and vocational education. Depending on the information provided, individuals are then categorised as having a ‘low’, ‘medium’ or ‘high’ level of education. Categorisation was based on the person with the highest educational attainment in the household.

##### Migration background

The determination of a child’s migration background was carried out in line with the operationalisation in the KiGGS study, according to which children and adolescents are considered to have a migration background if they themselves immigrated from another country or if at least one parent was not born in Germany or if both parents immigrated or had a non-German nationality [41]. For the analyses, children and adolescents without a migration background and with a one-sided migration background (through one parent) were grouped together and compared with children and adolescents with a two-sided migration background (through both parents), as people with no background and people with a one-sided background are more similar than people with a one-sided and a two-sided migration background.

The concept of ‘migration background’ has recently been criticised for not being sufficiently diverse [42]. Instead, it is recommended to stratify analyses according to individual variables such as country of birth, nationality, residence status or language skills. However, this was not possible in the present study due to insufficient case numbers in the single strata.

##### Family status

As in the KiGGS survey, parents were asked with whom the child lived. Families were then distinguished according to whether the child in question lived with both biological parents (nuclear family), with only one biological parent but without a partner (single-parent family) or with one biological parent and a partner (stepfamily). All other forms of family living arrangements, such as foster or adoptive families, living with grandparents or living permanently in an institution (e.g. a care home), were grouped together under the category ‘other’.

##### Urbanicity

Urbanicity (urban versus rural region) according to the INKAR classification (Indicators and Maps for Spatial and Urban Development) of the Federal Institute for Research on Building, Urban Affairs and Spatial Development [43] was added to the administrative dataset as an external variable.

##### Density of care at place of residence

In order to assess the care situation at the child’s place of residence, this study uses data from the KBV [44] on the nationwide care density (service providers per 100,000 inhabitants) for child and adolescent psychiatrists, medical psychotherapists, paediatricians and general practitioners. This information was also added to the administrative dataset as external variables. To structure the data, quintiles for each category of service provider were determined (1 = lowest 20 % and 5 = highest 20 % of the distribution). Each child was then assigned a quintile value for each category of provider, according to the density of care in their place of residence. Subsequently, mean values of the quintiles were computed. Mean values of 3 denote the expected value of the sample used as a basis; lower mean values denote a lower density of care in comparison, higher mean values denote a higher density of care in relation to the respective group of providers in the sample.

### 2.3 Statistical analysis

First, descriptives were calculated. Then, frequencies of parent-reported, ever medically or psychologically diagnosed ADHD were calculated, stratified by sociodemographic and care-related characteristics. The significance of group differences was tested using the Rao-Scott-Chi-Square test for complex samples. In addition, bivariate binary logistic regressions were used to calculate odds ratios (ORs) for the sociodemographic and care-related variables as predictors of parental report of the child’s administrative diagnosis of ADHD, as well as a multivariate logistic regression model that included all sociodemographic and care-related predictors simultaneously. All analyses were performed using the Stata/ SE 17.0 software package (Stata Corp., College Station, TX, USA, 2017), applying the svy procedure with population weights. Results of group comparisons with a significance level of *p* < 0.05 are considered significant.

## 3. Results

###  

#### Descriptive statistics

Approximately three quarters of the children and adolescents in the online sample were boys; the mean age at the time of the survey was 12.6 years (Table 1).

Just under a quarter of the children and young people were aged 7 to 10, just under a third were aged 11 to 13, a third were aged 14 to 17 and 3.5 % were aged 3 to 6. The proportion of 18- to 19-year-olds in the online sample was 7.5 %. Only three children were under the age of three at the time of the survey. Due to the small number of cases, this group was excluded from further analysis. Overall, just under two-thirds of the participating parents had a medium level of education, just over a quarter had a high level of education according to the CASMIN education classification, and about 10 % of the parents had a low level of education. Parents with a low level of education were thus underrepresented. The proportion of families with a both-sided migration background was 6.5 %. About two thirds of the participants came from urban areas and one third from rural areas. The mean of the quintiles for the density of medical psychotherapeutists, paediatricians and general practitioners was 3.0, while that for child and adolescent psychiatric care was 2.8. This means that the average care density for all mentioned groups of the service providers was in line with the expected mean values, with the exception of that of child and adolescent psychiatrists, where the lower mean value indicated a comparatively lower density of care.

#### Frequencies of a parent report of the child’s administrative diagnosis of ADHD, overall and by sociodemographic indicators

**Figure 1:**
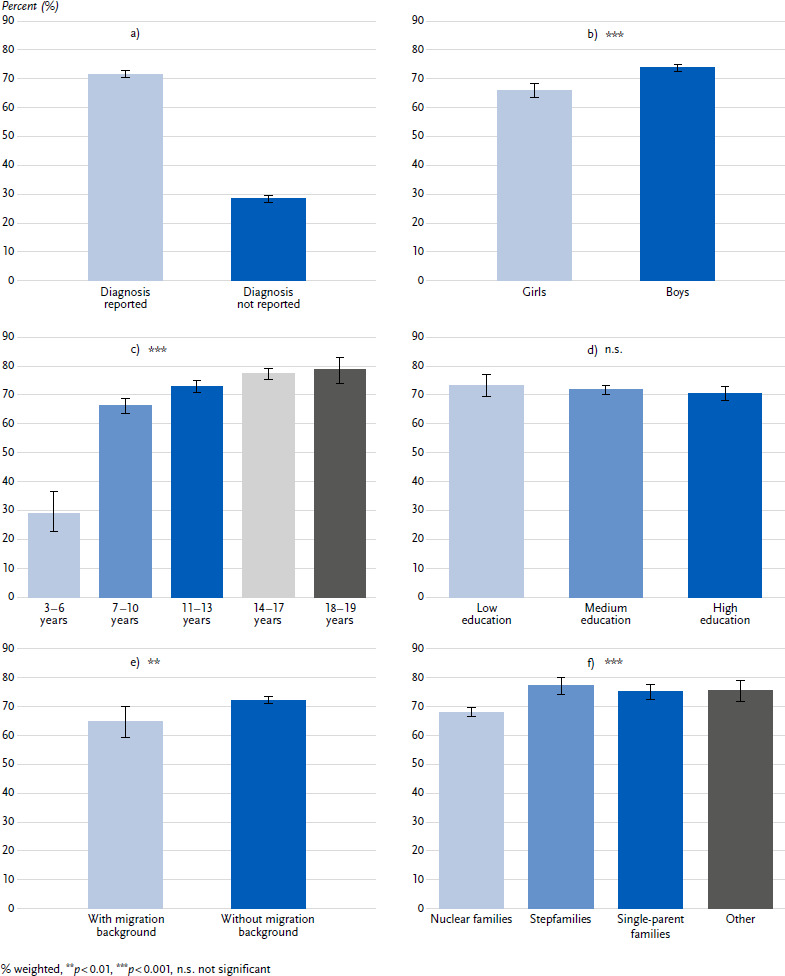
Parental report of an administrative ADHD diagnosis total, by gender, age groups, education, migration background, and family structure for 3- to 19-year-old children and adolescents (Figure 1a: *n* = 1,320 girls, *n* = 3, 888 boys; Figure 1b: *n* = 1,320 girls, *n* = 3,888 boys; Figure 1c: *n* = 1,320 girls, *n* = 3,888 boys; Figure 1d: *n* = 1,254 girls, *n* = 3,704 boys; Figure 1e: *n* = 1,279 girls, *n* = 3,759 boys; Figure 1f: *n* = 1,320 girls, *n* = 3,887 boys). Source: INTEGRATE-ADHD

Overall, 71.6 % of parents of children with a confirmed administrative diagnosis of ADHD in 2020 reported that their child had ever received a medical or psychological diagnosis of ADHD (Figure 1a).

An administrative diagnosis of ADHD was reported significantly more often for boys (73.7 %) than for girls (65.9 %) (Figure 1b). In children who were 3 to 6 years old at the time of the survey, only 29 % of parents reported an administrative diagnosis of ADHD for their child, compared to 66.1 % in 7- to 10-year-olds, 72.8 % in 11 - to 13-year-olds, 77.2 % in 14- to 17-year-olds and 78.7 % in 18- to 19-year-olds (Figure 1c). These differences prooved statistically significant. The analysis by age and gender showed that significant differences in the reporting frequency between the genders occurred primarily among the younger children (3- to 6-year-olds: girls 21.7 %, boys 31.7 %, *p* < 0.001; 7- to 10-year-olds: girls 56.4 %, boys 69.1 %, *p* < 0.001; 11 - to 13-year-olds: girls 64.1 %, boys 76.3 %, *p* < 0.001; 14- to 17-year-olds: girls 75.0 %, boys 77.8 %, *p* = 0.277; 18- to 19-year-olds: girls 80.9 %, boys 77.7 %, *p* = 0.539).

In contrast, there were no statistically significant differences in the frequency with which parents reported their child’s ADHD diagnosis in relation to educational status (Figure 1d). However, parents from families with a migration background were significantly less likely to report their child’s administrative diagnosis of ADHD (almost two thirds) compared to parents from families without a migration background (almost three quarters) (Figure 1e). Children from nuclear families with both biological parents were significantly less likely to have a reported ADHD diagnosis (68.1 %) than children from stepfamilies (77.4 %), single-parent families (75.1 %) or other family types (75.6 %) (Figure 1f). There were no significant differences in the reporting behaviour of parents according to region (rural vs. urban: *p* = 0.063) or according cording to the regional density of care (density of medical psychotherapists: *p* = 0.214; density of paediatricians and psychiatrists: *p* = 0.135; density of paediatricians: *p* = 0.226 and density of general practitioners: *p* = 0.823).

#### Bivariate and multivariate correlations

Table 2 shows the raw (bivariate) and adjusted (multivariate) odds ratios of the sociodemographic predictors under examination for predicting the likelihood of a parental diagnosis report in the epidemiological survey. In both the bivariate models and the overall multivariate model, male gender and older age of the child, no migration background, stepfamily, single-parent family or other family type were predictive of a parental report of an administrative diagnosis of ADHD in the child. Parental education, urbanicity or regional availability of medical-psychotherapeutic, child and adolescent psychiatric or paediatric care did not make a significant predictive contribution. The odds ratios of the significant predictors differed only slightly in the bivariate and multivariate analyses.

## 4. Discussion

The aim of this study was to analyse discrepancies between administrative and parent-reported data on ADHD diagnosis in children and adolescents, accounting for sociodemographic factors. The research question was motivated by the existing differences in prevalence and temporal trends between administrative and epidemiological survey-based data on ADHD diagnosis in children and adolescents in Germany. To our knowledge, this is the first study in Germany to combine administrative and parent-reported data on ADHD diagnosis in children and adolescents at the person level. The results provide information on parental reporting behaviour and its sociodemographic determinants and thus contribute to characterising and qualifying the relationship between administrative and epidemiological data on ADHD diagnosis.

In the epidemiological survey, a total of 71.6 % of parents reported their child’s administrative diagnosis of ADHD. The results thus show that a significant proportion of parents did not report their child’s administrative diagnosis of ADHD.

Roick and Waltersbacher [26] suggested that the differences in prevalence and in the time trend (increase in the administrative data and constant frequency of parent-reported diagnoses in KiGGS Wave 1) were due to a different approach to attention-deficit disorder without hyperactivity (ADHD). If service providers diagnosed a child with ADHD during the period of the KiGGS baseline study (2003 – 2006), they may have informed the parents about the diagnosis, but coded it according to the ICD criteria in the non-specific collective category F98.8 (Other unspecified behavioural and emotional disorders with onset in childhood and adolescence) [26]. In later years, encouraged by respective guideline recommendations [44, 45], health care providers may have aligned their diagnosis more closely with DSM-IV/-5 criteria and assigned the ICD-10 code F90.0 also for attention-deficit disorder without hyperactivity. That way, the diverging time trends of parent-reported and administrative diagnosis prevalences would have become explainable. Indeed, the case definition of the KiGGS study includes ADD according to DSM-IV/-5 criteria, whereas ADD diagnoses coded as ICD-10 F98.8 would not have been included in the administrative data analyses of the prevalence of hyperkinetic disorders (F90.0-9) for this period. The results of the present study, however, suggest that this hypothesis is not sufficient to explain the discrepancies between parent-reported and administrative diagnosis ADHD prevalences, but that other causes must be assumed, as reporting frequencies vary greatly according to sociodemographic characteristics.

For example, the administrative diagnosis of ADHD is reported less frequently for girls than for boys. It is unclear where this gender bias in reporting comes from. ADHD presents differently in girls and boys depending on their gender. In boys, overt hyperactive behaviour predominates, whereas in girls, less obvious inattention problems or more internalising symptoms such as easily distracted, disorganised, overstrained, or lacking effort or motivation often predominate [47, 48]. It has therefore been suggested that the disorder may be significantly underdiagnosed in girls [48, 49]. The different symptom presentation in girls and boys may also influence how parents assess the likelihood of their child having ADHD [50]. However, all children and adolescents in the present study had a confirmed administrative diagnosis of ADHD, including girls. The result may therefore be indicative of gender differences in communication between clinicians and patients or their parents, respectively, as parents can only report the diagnosis once they have been informed. It is conceivable that medical staff communicate differently with the parents of girls with ADHD than with the parents of boys with ADHD. The in-depth analysis showed that the gender differences in parental reporting of diagnosis to the detriment of girls only exist between the ages of 3 and 13 years. Why this occurs in the younger age groups – perhaps because the diagnosis in girls is less in line with parental expectations – deserves further investigation. Uncertainty in the diagnosis of girls or gender differences in the latency of diagnosis are not possible causes at least. Further research is needed in this area.

Parental reporting was lower in the lower age groups than in the higher ones. It was particularly low among parents of 3- to 6-year-olds, less than a third of whom reported their child’s administrative diagnosis of ADHD. It was also below average among 7- to 10-year-olds, although significantly higher. In KiGGS Wave 2, the decline in the prevalence of parent-reported ADHD diagnoses was found specifically for this age group [20, 24], though only in boys. The results of the present study suggest that the declines observed in KiGGS Wave 2 may be – at least in part – due to a parental reporting bias in this age group. However, this interpretation must be made with extreme caution, because due to the time lag between the administrative diagnosis and the interview date of at least 9 and a maximum of 32 months, some of the administrative diagnoses marked as confirmed for children who were 3 to 6 years old at the time of the interview may have been made at the age of 0 to 2 years. This raises concern, as ADHD cannot be diagnosed with certainty until after the age of three [51], and may have resulted in the diagnosis being communicated to parents less frequently or not being accepted by them. Indeed, using the INTEGRATE-ADHD data this proportion cannot be quantified with certainty, as for privacy reasons only age at the time of the survey could be recorded, but not date of birth.

Among children from nuclear families with both biological parents, the frequency of a parental diagnostic report was lower than among children from single-parent families and stepfamilies. This appears surprising, since previous analyses using the data from the KiGGS study suggested that children and adolescents from single-parent families and step-families displayed more hyperactivity symptoms and had an increased likelihood of a parent-reported ADHD diagnosis [20, 52–54]. It is conceivable that higher levels of distress are associated with greater willingness to report the diagnosis in an interview.

Parental education as a proxy for SES did not make a difference in terms of parental reporting behaviour. This is remarkable in view of the fact that the parent-reported prevalence of ADHD diagnosis in the KiGGS study was up to three times higher in children from families with a low SES than in children from families with a medium or high SES [20]. In INTEGRATE-ADHD, parents of children with a migration background were significantly less likely to report an administratively documented diagnosis of ADHD for their child than parents of children without a migration background. In each of the different waves of the KiGGS study, the frequency of parent-reported ADHD diagnoses was significantly lower for children with a migration background [20, 22, 23]. However, the frequency of children with clinically noticeable symptoms but without a parent-reported diagnosis of ADHD (so-called suspected cases) was equal to or higher than for children without a migration background [20]. Some authors suggest that ADHD may be underdiagnosed in children and adolescents with a migration background [55]. The results of the present study suggest that at least a part of the epidemiologically reported lower prevalence of ADHD in children and adolescents with a migration background is due to lower parental reporting in the survey. The extent to which language or cultural barriers may hinder communication between doctors and patients [56], or what other factors may influence willingness to report a diagnosis, should be investigated further.

The present study has several limitations and strengths. The time lag between the administrative data (base year 2020) and the online survey data (survey period October 2021 to August 2022) was a minimum of 9 months (31 December 2020 to the start of the online survey in early October 2021) and a maximum of 32 months (1 January 2020 to the end of the survey in August 2022). This may have affected the parents’ reporting behaviour in different ways. However, the administrative data were made available by DAK-Gesundheit only nine months after the end of the insurance year 2020, which in turn can be considered as very fast. In addition, the central indicator for ADHD in the KiGGS study, which was also used in the epidemiological survey of INTEGRATE-ADHD, asks whether a diagnosis had ever been made, i.e. the lifetime prevalence, whereas the administrative diagnoses in the INTEGRATE-ADHD sample represent the annual prevalence of the insurance year 2020. However, all children in the INTEGRATE-ADHD sample had a previous administrative diagnosis of ADHD, which should have been reported by parents in the epidemiological survey if they were aware of the diagnosis and willing to report it.

The design of the INTEGRATE-ADHD study cannot explain why parents did not report their child’s administrative diagnosis in the survey. Qualitative analyses would be helpful here. It is possible that parents were not informed of the diagnosis, did not understand it, or did not want to report it for a variety of reasons, such as fear of stigma, for example. Indeed, studies show that people with ADHD are subject to negative social perceptions [57, 58], which may affect the willingness to disclose a diagnosis of ADHD. Whether this also applies to parents of children with ADHD in the context of a scientific study is unknown, but cannot be ruled out here. In the invitation letter, parents were informed that the study would compare children with and without a diagnosis of ADHD and that their participation would be important even if they were not aware of an ADHD diagnosis of their child. INTEGRATE-ADHD also asked about the 12-month prevalence of ADHD and the use of a care service in 2020 due to the child’s ADHD. These indicators may retrospectively tighten the time gap between administrative diagnosis and survey to some extent and are the subject of further evaluations. After weighting, the study data can be considered to be approximately representative of the population, which increases the generalisability of the results. However, the weighting does not adjust for the specific ‘not missing at random’ non-response that exists when parents of children with ADHD do not participate because they are sure that their child has ADHD or for those who do not participate because they are sure that their child does not have ADHD. Finally, the project provides, for the first time in Germany, conjunct, individual-level linked administrative ADHD diagnostic data and epidemiological survey data.

In conclusion, this first evaluation of the data from the project INTEGRATE-ADHD suggests that at least some of the discrepancies between administrative ADHD diagnosis data and the epidemiological diagnosis frequencies determined by parental report in Germany can be attributed to the parent’s reporting behaviour and that the reporting frequencies vary according to different sociodemographic factors. Almost one third of parents did not report an administrative diagnosis of ADHD. This should be considered when contextualising both administrative and parent-reported ADHD diagnosis data in Germany in the future. Further analyses in the project INTEGRATE-ADHD will also focus on the verification of administrative and epidemiologically reported ADHD diagnoses through the guideline-based online ADHD diagnostics, which will has been carried out in a sub-sample. Further analyses of the determinants of parental reporting are underway or can be found in Pfeifer et al. [59], respectively. Possible reasons for the different reporting frequencies in the various sociodemographic strata could only be speculated upon. Due to the lack of precedence of the present research approach, further replicating and explanatory studies are needed.

## Key statement

- A proportion of 71.6 % of parents of children with a confirmed administrative diagnosis of ADHD report their child’s diagnosis in an epidemiological survey.
- The diagnosis is reported less frequently for girls, younger children, children with a migration background and children from nuclear families with both biological parents.
- There are no reporting differences according to parental education, urbanicity (urban/rural) and density of specialist care.

## Figures and Tables

**Table 1:** Characteristics of the online sample of the INTEGRATE-ADHD project. Source: INTEGRATE-ADHD

Online sample INTEGRATE-ADHD			
	*n*	% (95 % CI)	Mean (SE)
**Gender**			
Girls	1,386	25.9 (24.7 – 27.1)	–
Boys	4,075	74.1 (72.9 – 75.3)	–
**Mean age^a^**	5,461	–	12.6 (0.05)
**Age group^a^**			
0 – 2 years	3	0.1 (0.0 – 0.2)	–
3 – 6 years	167	3.5 (3.0 – 4.0)	–
7 – 10 years	1,351	24.3 (23.1 – 25.4)	–
11 – 13 years	1,827	31.2 (30.0 – 32.5)	–
14 – 17 years	1,770	33.4 (32.2 – 34.8)	–
18 – 19 years	343	7.5 (6.8 – 8.3)	–
**Parental education (CASMIN)^b^**			
Low	560	10.4 (9.6 – 11.3)	–
Medium	3,271	63.2 (61.9 – 64.5)	–
High	1,355	26.4 (25.1 – 27.6)	–
**Migration background (two-sided)**			
No	4,948	93.5 (92.7 – 94.1)	–
Yes	332	6.5 (5.9 – 7.3)	–
**Urbanicity**			
Urban	3,431	63.6 (62.3 – 64.9)	–
Rural	1,949	36.4 (35.1 – 37.7)	–
**Density of care^c^**			
Medical psychotherapists	5,380	–	3.0 (0.02)
Child and adolescent psychiatrists	5,380	–	2.8 (0.02)
Paediatrians	5,380	–	3.0 (0.02)
General practitioners	5,380	–	3.0 (0.02)

CI = confidence interval, SE = standard error, CASMIN = Comparative Analysis of Social Mobility in Industrial Nations % = weighted, n = unweighted

^a^At the time of survey

^b^Person with highest educational qualification in the household

^c^Mean value of the quintiles of the regional ratio per 100,000 inhabitants in the spatial planning region of the child’s place of residence

**Table 2:** Crude and adjusted odds ratios for parental report of administrative diagnosis of ADHD in children and adolescents aged 3 to 19 years. Source: INTEGRATE-ADHD

	OR (95 % CI)	AOR (95 % CI)
**Gender**		
Girls	Ref.	Ref.
Boys	**1.45 (1.26 – 1.67)** *n* = 5,208	**1.54 (1.33 – 1.79)**
**Mean age**	**1.13 (1.10 – 1.15)** *n* = 5,208	**1.12 (1.10 – 1.15)**
**Parental education (CASMIN)**		
Low	1.07 (0.86 – 1.34)	1.04 (0.83 – 1.31)
Medium	Ref.	Ref.
High	0.94 (0.81 – 1.10) *n* = 4,958	0.98 (0.83 – 1.14)
**Migration background (two-sided)**		
No	**1.41 (1.10 – 1.82)**	**1.51 (1.16 – 1.98)**
Yes	Ref. *n* = 5,038	Ref.
**Family status**		
Nuclear families	Ref.	Ref.
Stepfamilies	**1.60 (1.31 – 1.94)**	**1.46 (1.19 – 1.80)**
Single-parent families	**1.41 (1.18 – 1.68)**	**1.27 (1.06 – 1.54)**
Other	**1.45 (1.16 – 1.81)** *n* = 5,207	**1.37 (1.06 – 1.76)**
**Urbanicity**		
Rural	0.88 (0.77 – 1.01)	0.89 (0.76 – 1.05)
Urban	Ref. *n* = 5,129	Ref.
**Density of care^a^**		
Medical psychotherapists	1.03 (0.98 – 1.08) *n* = 5,129	1.02 (0.96 – 1.09)
Child and adolescent psychiatrists	1.03 (0.99 – 1.08) *n* = 5,129	1.02 (0.97 – 1.07)
Paediatrians	1.03 (0.98 – 1.08) *n* = 5,129	1.00 (0.94 – 1.07)
General practitioners	1.01 (0.96 – 1.05) *n* = 5,129	0.99 (0.93 – 1.05) *n* = 4,883

OR = Odds Ratio, AOR = Adjusted Odds Ratio, CI = confidence interval, CASMIN = Comparative Analysis of Social Mobility in Industrial Nations All regressions weighted, n = unweighted, bold = statistically significant

^a^ Quintiles of regional ratio per 100,000 inhabitants in the spatial planning region of the child’s place of residence
